# Multigene Phylogeny, Diversity and Antimicrobial Potential of Endophytic Sordariomycetes From *Rosa roxburghii*

**DOI:** 10.3389/fmicb.2021.755919

**Published:** 2021-11-29

**Authors:** Hong Zhang, Tian-Peng Wei, Lin-Zhu Li, Ming-Yan Luo, Wei-Yu Jia, Yan Zeng, Yu-Lan Jiang, Guang-Can Tao

**Affiliations:** ^1^Department of Plant Pathology, College of Agriculture, Guizhou University, Guiyang, China; ^2^Guizhou Academy of Testing and Analysis, Guiyang, China; ^3^Food Safety and Nutrition (Guizhou) Information Technology Co., Ltd., Guiyang National High-Tech Industrial Development Zone, Guiyang, China

**Keywords:** molecular phylogeny, alpha diversity, lifestyle diversity, intracellular metabolites, extracellular metabolites, *Hypomontagnella monticulosa*, *Nigrospora sphaerica*

## Abstract

*Rosa roxburghii* Tratt. is widely applied in food, cosmetics, and traditional medicine, and has been demonstrated to possess diverse bioactivities. Plant endophytic fungi are important microbial resources with great potential for application in many fields. They not only establish mutualistic symbiosis with host plants but also produce a variety of bioactive compounds. Therefore, in the present study, endophytic fungi were isolated from *R. roxburghii*, the diversity and antimicrobial activities were evaluated. As a result, 242 strains of endophytic Sordariomycetes were successfully isolated. Multigene phylogenetic analyses showed that these isolates included eight orders, 19 families, 33 genera. The dominant genera were *Diaporthe* (31.4%), *Fusarium* (14.4%), *Chaetomium* (7.9%), *Dactylonectria* (7.0%), *Graphium* (4.5%), *Colletotrichum* (4.1%), and *Clonostachys* (4.1%). For different tissues of *R. roxburghii*, alpha diversity analysis revealed that the diversity of fungal communities decreased in the order of root, fruit, stem, flower, leaf, and seed, and *Clonostachys* and *Dactylonectria* exhibited obvious tissue specificity. Meanwhile, functional annotation of 33 genera indicated that some fungi have multitrophic lifestyles combining endophytic, pathogenic, and saprophytic behavior. Additionally, antimicrobial activities of endophytic Sordariomycetes against *Lasiodiplodia theobromae*, *Botryosphaeria dothidea*, *Colletotrichum capsici*, *Pyricularia oryzae*, *Rhizoctonia solani*, *Fusarium oxysporum*, *Pseudomonas syringae*, *Pantoea agglomerans*, *Staphylococcus aureus*, *Bacillus subtilis*, *Escherichia coli*, and *Pseudomonas aeruginosa* were screened. Dual culture test assays showed that there were 40 different endophytic species with strong inhibition of at least one or moderate inhibition of two or more against the 12 tested strains. The results from the filter paper diffusion method suggested that extracellular metabolites may be more advantageous than intracellular metabolites in the development of antimicrobial agents. Eleven isolates with good activities were screened. In particular, *Hypomontagnella monticulosa* HGUP194009 and *Nigrospora sphaerica* HGUP191020 have shown promise in both broad-spectrum and intensity. Finally, some fungi that commonly cause disease have been observed to have beneficial biological activities as endophytic fungi. In conclusion, this study showed the species composition, alpha diversity, and lifestyle diversity of endophytic Sordariomycetes from *R. roxburghii* and demonstrated these isolates are potential sources for exploring antimicrobial agents.

## Introduction

Endophytic fungi are highly diverse taxa of microorganisms that inhabit asymptomatically in healthy tissues of living plants (Jia et al., 2016), and are currently not completely explored. They have increasingly received much attention due to their biological activities, such as antifungal, antibacterial, antivirus, insecticides, antioxidant, cytotoxic, alpha-glucosidase inhibitory, anti-inflammation, antidiabetic, and anticancer properties (Deshmukh et al., 2015; Zhang et al., 2019; Fernando et al., 2020; Manganyi and Ateba, 2020; Pal et al., 2020; Peng et al., 2020; Rahaman et al., 2020; Agrawal et al., 2021). In the field of plant protection, fungal endophytes are considered as one of the most important sources of potential biocontrol agents because of antimicrobial, antivirus, insecticides, and the alleviation of abiotic stresses (Park et al., 2017; Wen et al., 2019). They can reduce disease severity and pathogen biomass in the host plant via nutrient and space competition and producing various antagonistic secondary metabolites (Rojas et al., 2020; Xie et al., 2020). These metabolites showed inhibitory activity against the spore germination of the pathogens and were also able to cause morphological changes, reduced the expression of the genes involved in mycelial growth and virulence, and induced defense-related genes (Park et al., 2015; Sánchez-Fernández et al., 2020). So, biological control based on fungal endophytes and their functional secondary metabolites has broad application prospects. Meanwhile, endophytic fungi are also well known as a source of antimicrobial agents for pharmaceutical development.

Sordariomycetes, the second-largest class of the phylum Ascomycota, including 28 orders, 90 families and 1,344 genera, 829 uncertain genera, and over 10,000 species, is also the dominant community member of endophytic fungi from various plants (Fonseca-García et al., 2016; Maharachchikumbura et al., 2016). Some species of Sordariomycetes have also been considered important biocontrol agents, e.g., *Trichoderma* spp., *Hypoxylon anthochroum* and *Induratia alba* (syn. *Muscodor albus*), and others produce a variety of bioactive compounds that are important to the biotechnology industries (Leylaie and Zafari, 2018; Macías-Rubalcava et al., 2018; Tilocca et al., 2020).

*Rosa roxburghii* Tratt., an economically important crop of homologous medicine and food, is characterized by a notably high content of vitamin C, up to 1,000 mg/100 g (Xu et al., 2019). The wild *R. roxburghii* is mainly distributed in Southwest China, while it is only domesticated and cultivated on a large scale in Guizhou Province, China. In recent years, it was widely applied in food and cosmetics for its nutritional and health benefits (Yang et al., 2020). Its root, leaf, and fruit have been used as traditional medicinal materials in the treatment of several diseases, including strengthening the spleen, dyspepsia, enteritis, and scurvy (Wang et al., 2018). Modern pharmacological studies have demonstrated that some components of *R. roxburghii* exhibited several biological activities, such as excellent hypoglycemic and hypolipidemic effects, enhancing immunity, antioxidation, and anti-tumor activities (Wang et al., 2018).

Given the high dietary and medicinal value of *R. roxburghii* and there have been few investigations of endophytes isolated from this plant, in this study, we selected it as a source plant to isolate culturable endophytes through different media. We further assessed the diversity and antimicrobial potential of endophytic Sordariomycetes. This work will aid in the search for novel endophytic species or strains that possess valuable bioactive compounds and also set the basis for the foundation of promoting growth and improving immunity and stress resistance of *R. roxburghii.*

## Materials and Methods

### Isolation and Identification

#### Sample Collection and Endophyte Isolation

From April to August 2020, Healthy *R. roxburghii* tissues were collected from Guiyang City (27°4′50″ N, 106°29′50″ E) and Liupanshui City (25°52′52″ N, 104°33′59″ E), Guizhou Province, China. Endophytic fungi were isolated from the sample blocks according to the method described by Liu et al. (2015). To isolate as many endophytes as possible, six different media, including potato dextrose agar (PDA), oatmeal agar (OA), malt extract agar (MEA), Czapek Dox Agar (CDA), water agar (WA), and synthetic low nutrient agar (SNA) medium, were used for isolation.

#### DNA Extraction, Polymerase Chain Reaction Amplification, and Sequencing

DNA was extracted from fresh mycelia grown on PDA using the Fungal gDNA Isolation Kit (BW-GD2416, Biomiga, China), following the manufacturer’s instructions. The extracted DNA was used as the template for the polymerase chain reaction (PCR). The primers and PCR reaction conditions were given in Table 1. The amplified PCR products were directed to Sangon Biotech (Shanghai) Co., Ltd. (Shanghai, China) for sequencing.

**TABLE 1:** Details of genes/loci with PCR primers and PCR profiles.

**Gene/Loci**	**PCR primers (forward/reverse)**	**PCR condition**
**ITS**	ITS5/ITS4	94°C: 3 min, 35 cycles (94°C: 30 s, 55°C: 30 s, 72°C: 45 s), 72°C: 10 min, final hold at 4°C
**LSU**	LROR/LR5	94°C: 3 min, 35 cycles (94°C: 30 s, 55°C: 30 s, 72°C: 45 s), 72°C: 10 min, final hold at 4°C
**TUB**	Bt2a/Bt2b	95°C: 5 min, 35 cycles (94°C: 1 min, 55°C: 1 min, 72°C: 2 min), 72°C: 10 min, final hold at 4°C

#### Multigene Phylogenetic Analyses

Endophytic fungi were identified by protein-coding and ribosomal gene sequences. All forward and reverse sequences were used to create consensus sequences by BioEdit v. 7.0.9.0 (Hall, 1999), and BLASTn searched in NCBI to identify genus-level taxonomic status. Multigene phylogenetic analyses were performed by Maximum Likelihood (ML) and Bayesian inference (BI) methods. The sequences were aligned with MAFFT v. 7 (Katoh et al., 2019). Alignments were adjusted manually in BioEdit v. 7.0.9.0 (Hall, 1999) and concatenated in PhyloSuite v. 1.2.2 (Zhang D. et al., 2020). The best-fit partition models were inferred using ModelFinder (Kalyaanamoorthy et al., 2017), ML was conducted using IQ-TREE (Nguyen et al., 2014), BI was carried out using MrBayes 3.2.6 (Ronquist et al., 2012), and they have been integrated into PhyloSuite (Zhang D. et al., 2020).

### Diversity Indices and Functional Annotations Analysis

#### Dominant Taxa

A taxon is defined as dominant if *P_*i*_* > Camargo’s index (1/*S*), where *S* represents species richness, which is the number of fungal taxa, *P*_*i*_ is calculated as the number of isolates (*N*_*i*_) that belong to a certain taxon (*i*) divided by the total number of isolates (*N*) (Camargo, 1992; Kusari et al., 2013).

#### Alpha Diversity

To quantify the alpha diversity of endophytic Sordariomycetes from *R. roxburghii* in different tissues, the Shannon-Wiener index (*H′*), Simpson index (*D*), Margalef index (*dM*), and Pielou evenness index (*J*) were calculated using the following equations, respectively (Kusari et al., 2013; Li et al., 2016):


$$H^{\prime }\,=-\sum_{i\,=1}^{S}\left(P_{i}\right)\,\left(\ln P_{i}\right)$$

$$D\,=1-\sum_{i\,=1}^{S}{\left(P_{i}\right)}^{2}$$

d⁢M⁢=(S-1)/ln⁡N

J=H/′H,′m⁢a⁢xHm⁢a⁢x′=lnS

Where, *P*_*i*_, *S*, and *N* as described above.

#### Lifestyle Diversity

Lifestyle status for culturable fungi was predicated using the FUNGuild database^1^. Functional annotation of fungi at the genus level was considered reasonable (Nguyen et al., 2016).

### Antimicrobial Activity

#### Tested Strains

Twelve tested strains were used to evaluate antimicrobial potential of endophytic Sordariomycetes. Six phytopathogenic fungi (*Lasiodiplodia theobromae*, *Botryosphaeria dothidea*, *Colletotrichum capsici*, *Pyricularia oryzae*, *Rhizoctonia solani*, and *Fusarium oxysporum*), two phytopathogenic bacteria (*Pseudomonas syringae* pv. *actinidiae*, and *Pantoea agglomerans*), two Gram-positive bacteria [*Staphylococcus aureus*, ATCC 6538; and *Bacillus subtilis*, CMCC (B) 63501], and two Gram-negative [*Escherichia coli*, CMCC (B) 44102; and *Pseudomonas aeruginosa*, ATCC 27853].

#### Preliminary Antimicrobial Screening

Preliminary screening assays for antifungal activity were performed following the method of Singh et al. (2020). Briefly, mycelial plugs (6 mm) of tested fungi were inoculated at the center of PDA plates, the plugs (6 mm) of endophyte strains were placed at the edge of PDA plates. All plates were incubated for 2–7 days at 28 ± 1°C. Thereafter, the width of inhibitory bands (*I*) between tested fungi and endophytes was measured, with three replicates. 0 (*I* = 0), no inhibition; 1 (0 < *I* ≤ 1 mm), weak inhibition; 2 (1 mm < *I* ≤ 3 mm), moderate inhibition; 3 (*I* > 3 mm), strong inhibition (Zhao et al., 2019).

Preliminary antibacterial screening assays were carried out by inoculation of endophytic fungi with mycelial plugs. Mycelial plugs (6 mm) were placed symmetrically on nutrient agar (NA) plates, which were already coated with tested bacteria. The width of inhibitory bands (*I*) between tested bacteria and endophytes was measured after culturing for 48 h at 25 ± 1°C for phytopathogenic bacteria, 35 ± 1°C for other bacteria, with three replicates. 0 (*I* ≤ 1 mm), no inhibition; 1 (1 mm < *I* ≤ 2 mm), weak inhibition; 2 (2 mm < *I* ≤ 10 mm), moderate inhibition; 3 (*I* > 10 mm), strong inhibition (Gashgari et al., 2016).

#### Extracellular and Intracellular Metabolite Extraction

Following molecular identification and preliminary screening, strains would be selected for further study by the following principles: (1) with the best inhibition effect in the same species; (2) strong inhibition of at least one or moderate inhibition of two or more against the 12 tested strains.

Strains that screened according to the above principles were inoculated separately into 250 mL Erlenmeyer flasks with 100 mL of potato dextrose broth (potato: 200 g/L; dextrose: 20 g/L; natural pH). These flasks were cultured in a rotating incubator (28 ± 1°C, 220 rpm) for 7–10 days. The fermentation broth was then separated from the mycelium by vacuum filtration or high-speed centrifugation (12,000 rpm). The culture broth was extracted three times with the same volume of ethyl acetate (EtOAc). The mycelium was extracted with 150 mL of methanol (MeOH) under ultrasonication for 1 h. The suspension was filtered and the mycelium was discarded. Extracts were concentrated under reduced pressure using a rotary vacuum evaporator at 50°C until constant weight and dissolved in dimethyl sulfoxide (DMSO) to prepare 20 mg/mL of extracellular and intracellular metabolites. All crude extracts were stored at −20°C.

#### Re-screening of Antifungal Activity Assay

Antifungal activity of extracellular and intracellular metabolites was re-screened according to Hu et al. (2017). Mycelial plugs (6 mm) of the tested fungi were inoculated on PDA plates, 2 cm from the edge of the plates. Immediately afterward, sterile filter paper disks (6 mm) were placed at equal distances from the opposite edge, impregnated with 10 μL of crude extract (20 mg/mL). DMSO was used as the negative control. All plates were incubated at 28 ± 1°C for 2–7 days. Radial growth of tested strains was measured. The percentage inhibition (%) = (R_1_–R_2_)/R_1_ × 100% (Hajieghrari et al., 2008), where R_1_ is radial growth measurement of the tested strains in control, R_2_ is radial growth of the tested strains in the presence of metabolites. Similarly, the minimum inhibitory concentration (MIC) tests were performed.

#### Re-screening of Antibacterial Activity Assay

The filter paper disk diffusion method was used to re-screen the antibacterial activity of secondary metabolites (Rjeibi et al., 2020). Sterile filter paper disks (6 mm) were placed on the center of NA plates that had been coated with the tested bacteria. Then the disks were impregnated with 10 μL of metabolites (20 mg/mL). DMSO was applied as the negative control. Incubation conditions were the same as the preliminary screening, and the diameter of the inhibition zone was measured, with three replicates. Similarly, the MICs were determined.

### Data Analysis

Statistical analyses were conducted using Data Processing System (DPS v9.50). Data were analyzed by ANOVA, followed by comparisons of means using the LSD test (*P* < 0.05).

## Results

### Endophytic Sordariomycetes Identification

**FIGURE 1:**
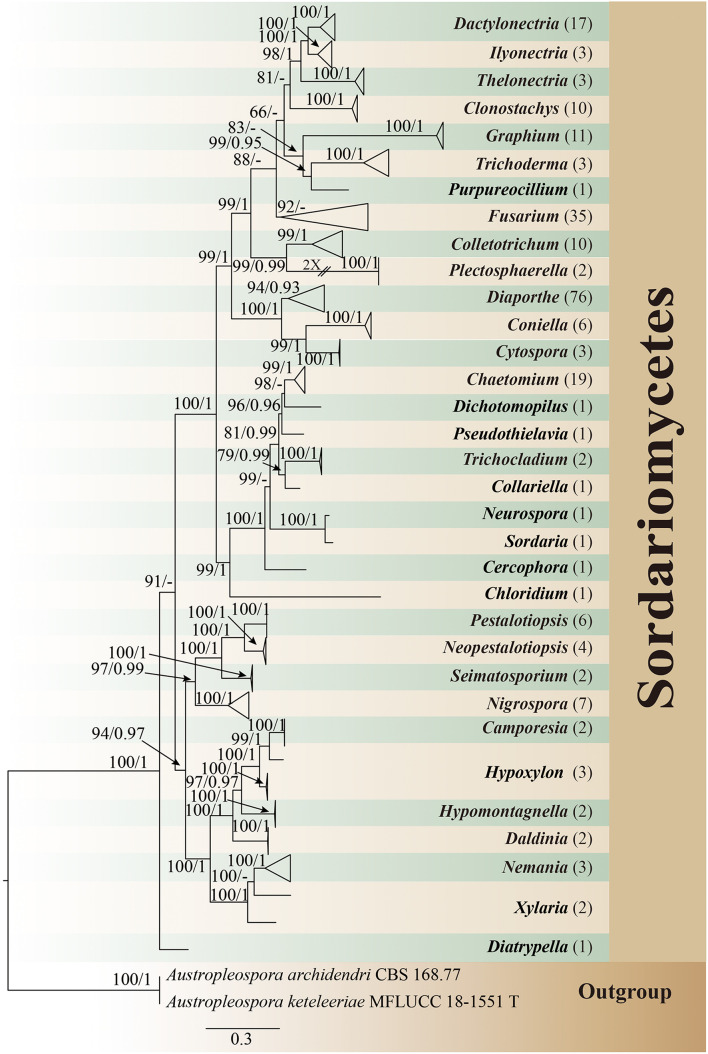
Phylogram generated from maximum likelihood analysis based on combined ITS, LSU, and TUB sequence data of endophytic Sordariomycetes in this study. *Austropleospora archidendri* (CBS 168.77) and *A. keteleeriae* (MFLUCC 18-1551) are used as the outgroup taxa. Bootstrap support values for ML greater than 50%, and Bayesian posterior probabilities greater than 0.90 are given near nodes (BS/PP), respectively. The number in brackets represents the number of endophytic isolates. T, type.

All isolates were identified through multigene phylogenetic analysis of the combined internal transcribed spacer (ITS), 28S large subunit rDNA (LSU), and beta-tubulin (TUB). A total of 242 isolates of endophytic Sordariomycetes were obtained and identified from the root, stem, leaf, flower, fruit, and seed segments of *R. roxburghii*. The phylogenetic relationship of these isolates was shown in Figure 1, containing eight orders, 19 families, 33 genera. One hundred and ninety of these (78.5%) were identified at the species level, covering 69 confirmed species. The rest (21.5%) were characterized at the genus level, including 30 unconfirmed species, possibly belonging to new taxa.

### Diversity of Endophytic Sordariomycetes

#### Dominant Taxa

**FIGURE 2:**
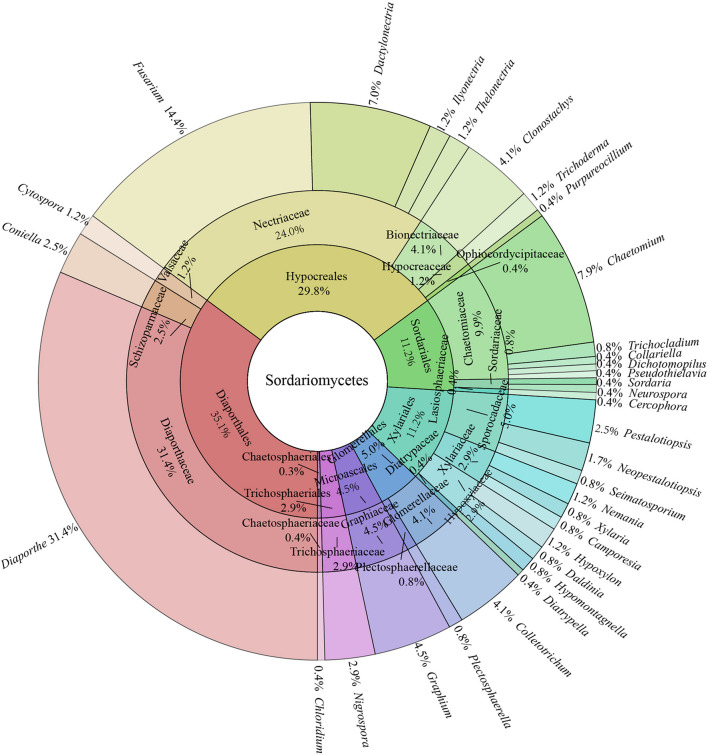
Species composition of endophytic Sordariomycetes from *Rosa roxburghii*.

In all isolates of endophytic Sordariomycetes, the Camargo’s index (1/*S*) at the order, family, and genus level were 0.125, 0.053, and 0.030, respectively. Therefore, the dominant orders were Diaporthales (35.1%) and Hypocreales (29.8%); the dominant families were Diaporthaceae (31.4%), Nectriaceae (29.8%), and Chaetomiaceae (9.9%); and the dominant genera were *Diaporthe* (31.4%), *Fusarium* (14.4%), *Chaetomium* (7.9%), *Dactylonectria* (7.0%), *Graphium* (4.5%), *Colletotrichum* (4.1%), and *Clonostachys* (4.1%) details as in Figure 2.

#### Alpha Diversity

**FIGURE 3:**
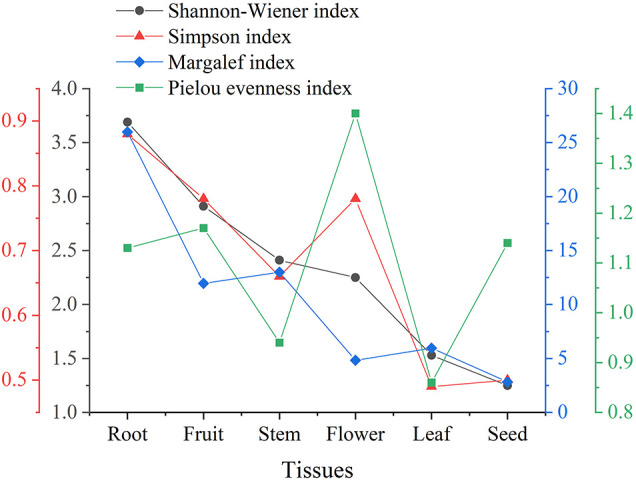
Alpha diversity of endophytic Sordariomycetes from *Rosa roxburghii*.

The species diversity can be assessed by the Shannon-Wiener index (*H’*) and Simpson index (*D*). In general, the higher the *H’* (usually ranging from 1.5 to 4.5) and the closer the *D* to 1, the more intensified heritable variation and the better the ability to adapt to micro-environmental changes. Margalef index (*dM*) can reflect the richness of endophytic fungi species. The larger the *dM*, the more abundant the endophytic fungal species. Additionally, the Pielou evenness index (*J*) can reflect the evenness of species (Li et al., 2016). Typically, the trends of *H’* and *dM* remained consistent. However, as illustrated in Figure 3, the value of *D* was higher in floral tissues than in stem tissues, which might be attributed to the higher values of *J*. The endophytic fungi in floral tissues (five species, six isolates) were much lower than in stem tissues (13 species, 72 isolates) in both species and number. In cases of poor diversity, as in seed tissue, the Pielou evenness index had little effect on the other indices. Taken together, the diversity of endophytic Sordariomycetes from different tissues decreased in the order of root, fruit, stem, flower, leaf, and seed. Notably, both 10 isolates of *Clonostachys* and 17 isolates of *Dactylonectria* were present only in root tissues.

#### Lifestyle Diversity

**FIGURE 4:**
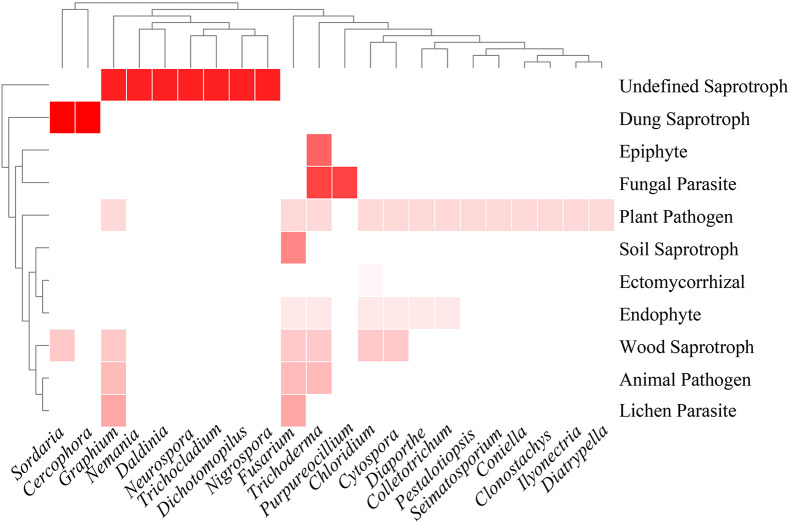
Heatmap of functional annotations of culturable fungi. Different shades of red indicate different fungal lifestyles.

Thirty-three genera of Sordariomycetes were analyzed for functional annotation in the FUNGuild database. Of these, no information was obtained in 11 genera, including *Plectosphaerella*, *Dactylonectria*, *Thelonectria*, *Chaetomium*, *Collariella*, *Pseudothielavia*, *Xylaria*, *Hypomontagnella*, *Camporesia*, *Hypoxylon*, and *Neopestalotiopsis.* Functional annotations of other genera were as described in Figure 4. Plant pathogen and saprotroph (wood saprotroph, soil saprotroph, dung saprotroph, and undefined saprotroph) dominated the fungal communities, followed by endophytic fungi (6/22). Five genera, namely *Chloridium*, *Cytospora*, *Fusarium*, *Trichoderma*, and *Graphium*, were found to have three or more lifestyles.

### Antimicrobial Activity

#### Preliminary Antimicrobial Screening

**FIGURE 5:**
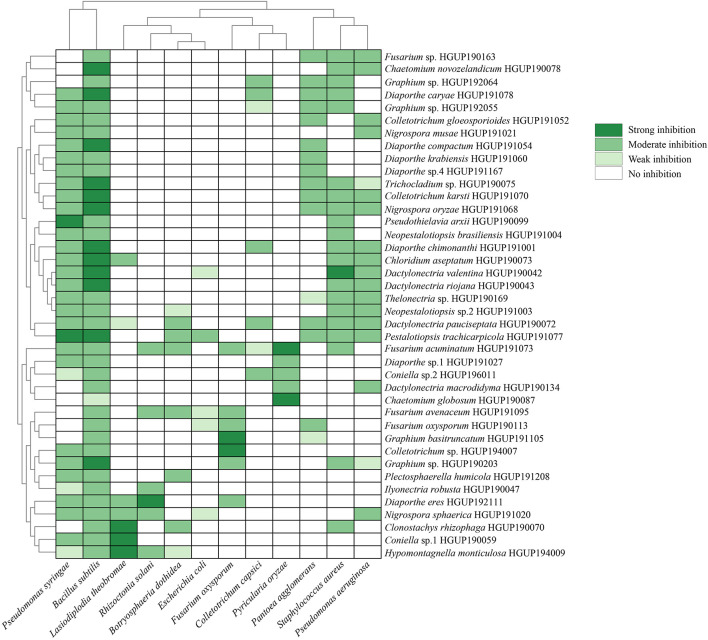
Heatmap of antimicrobial activity spectra of endophytic Sordariomycetes against the tested strains.

In this study, 242 strains of endophytic Sordariomycetes were first screened for antimicrobial activity. There were 40 strains from different species that exhibited at least one strong inhibition or two or more moderate inhibition against the 12 tested strains, as presented in Figure 5. Almost all screened isolates (39/40) showed moderate and above inhibition against *Bacillus subtilis*. The same effect was observed for 70% (28/40) of these against *Pseudomonas syringae* pv. *actinidiae*. In addition, *Pantoea agglomerans*, *Staphylococcus aureus*, and *Pseudomonas aeruginosa* were effectively inhibited by nearly half of the selected isolates. Altogether, the results indicated that many species of endophytic Sordariomycetes exhibited broad-spectrum antimicrobial activity, such as *Hypomontagnella monticulosa* HGUP194009, *Nigrospora sphaerica* HGUP191020, and *Pestalotiopsis trachicarpicola* HGUP191077.

#### Re-screening of Antifungal Activity

**FIGURE 6:**
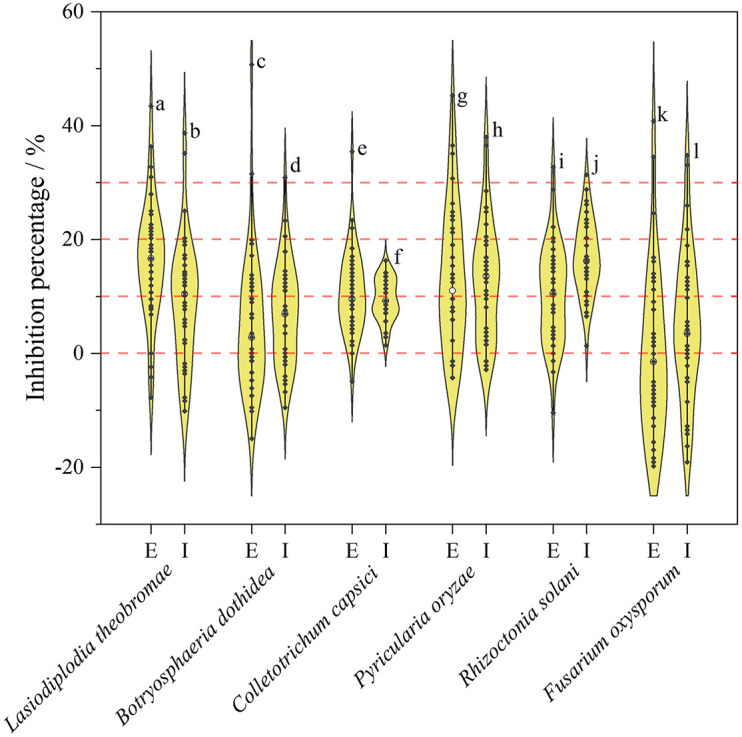
Violin plot of antifungal activity of endophytic Sordariomycetes. The width of the violin plot represents the number of endophytic fungi at the corresponding inhibition rate. **a**: HGUP194009 (43.45 ± 1.03%); **b**: HGUP190070 (38.69 ± 2.06%); **c**: HGUP194009 (50.72 ± 2.05%); **d**: HGUP190163 (30.87 ± 2.37%); **e**: HGUP190087 (35.46 ± 1.23%); **f**: HGUP191003 (16.31 ± 1.23%); **g**: HGUP196011 (45.30 ± 2.19%); **h**: HGUP191001 (38.00 ± 1.26%); **i**: HGUP194009 (32.68 ± 2.26%); **j**: HGUP190073 (31.37 ± 1.96%); **k**: HGUP192111 (40.80 ± 2.12%); **l**: HGUP190099 (34.81 ± 0.61%). E, Extracellular metabolites; I, Intracellular metabolites.

Inhibition rates of secondary metabolites were displayed as a violin plot (Figure 6). Most endophytic fungi, either extracellular or intracellular metabolites, exhibited lower inhibition rates (<30%). As observed from the trends of Figure 6, extracellular metabolites may have an advantage over intracellular metabolites in the development of antimicrobial agents. Among the extracellular products, *H. monticulosa* HGUP194009 exhibited significant inhibitory activity against *Lasiodiplodia theobromae* and *Botryosphaeria dothidea*, with 43.45 ± 1.03% and 50.72 ± 2.05% inhibition, respectively. In addition, *Coniella* sp. 2 HGUP196011 (45.30 ± 2.19%) and *Diaporthe eres* HGUP192111 (40.80 ± 2.12%) also displayed obvious inhibitory effects against *Pyricularia oryzae* and *Fusarium oxysporum*, in that order. Of note is that some secondary metabolites were observed in this study that may promote the growth of phytopathogenic fungi.

#### Re-screening of Antibacterial Activity

**FIGURE 7:**
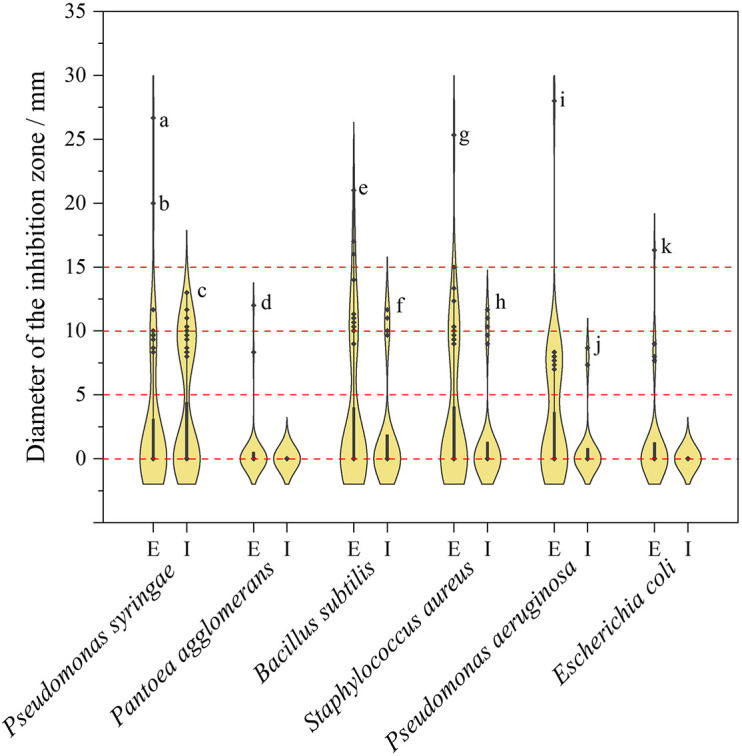
Violin plot of antibacterial activity of endophytic Sordariomycetes. The width of the violin plot represents the number of endophytic fungi at the corresponding inhibition diameter. **a**: HGUP191078 (26.67 ± 1.53 mm); **b**: HGUP191020 (20.00 ± 2.00 mm); **c**: HGUP190043 (13.00 ± 1.00 mm); **d**: HGUP191020 (12.00 ± 1.00 mm); **e**: HGUP191020 (21.00 ± 1.00 mm); **f**: HGUP190099 (11.67 ± 0.58 mm); **g**: HGUP191020 (25.33 ± 1.53 mm); **h**: HGUP191003 (11.67 ± 0.58 mm); **i**: HGUP191020 (28.00 ± 1.00 mm); **j**: HGUP191105 (8.67 ± 1.53 mm); **k**: HGUP191020 (16.33 ± 1.53 mm). E, Extracellular metabolites; I, Intracellular metabolites.

Violin plot of inhibition diameter of endophytic Sordariomycetes represented in Figure 7. The diameter of the inhibition zone is less than 6 mm, which means no antibacterial activity because the diameter of the sterile filter paper disks is 6 mm. As can be derived from Figure 7, the antibacterial activity of the extracellular metabolites was superior to that of the intracellular metabolites, which was similar to the results of antifungal activity. The diameters distributed predominantly between 8 and 12 mm, except for those without antibacterial activity, which suggested that most metabolites of endophytic fungi had moderate or below antibacterial activity. Among the extracellular metabolites, *Nigrospora sphaerica* HGUP191020 showed the strongest inhibitory activity against *Pantoea agglomerans*, *Bacillus subtilis*, *Staphylococcus aureus*, *Pseudomonas aeruginosa*, and *Escherichia coli* compared with other endophytic strains. The isolate had a significant inhibitory effect against *Pseudomonas syringae* as well, with an inhibition diameter of 20.00 ± 2.00 mm. Additionally, *Diaporthe caryae* HGUP191078 also displayed strong inhibitory (26.67 ± 1.53 mm) against *Pseudomonas syringae*.

#### Determination of the Minimum Inhibitory Concentration

To evaluate the antimicrobial potential of the extracellular metabolites, MICs of the isolates that exhibited a strong and broad-spectrum inhibition in the re-screening assays were determined, as depicted in Table 2.

**TABLE 2:** The minimum inhibitory concentration (MIC) of extracellular metabolites against 12 tested strains.

**Species**	**Strain no.**	**MIC concentration (mg/ml)**					
		***Lasiodiplodia theobromae***	***Botryosphaeria dothidea***	***Colletotrichum capsici***	***Pyricularia oryzae***	***Rhizoctonia solani***	***Fusarium oxysporum***
*Hypomontagnella monticulosa*	HGUP194009	2.50	1.25	–	–	5.00	10.00
*Nigrospora sphaerica*	HGUP191020	10.00	–	–	10	–	–

**Species**	**Strain no.**	***Pseudomonas syringae***	***Pantoea agglomerans***	***Staphylococcus aureus***	***Bacillus subtilis***	***Escherichia coli***	***Pseudomonas aeruginosa***

*Hypomontagnella monticulosa*	HGUP194009	0.31	10.00	5.00	2.50	–	5.00
*Nigrospora sphaerica*	HGUP191020	1.25	1.25	2.50	5.00	1.25	5.00

## Discussion

Although tremendous breakthroughs have been made in fungal species identification through molecular techniques, identification remains a considerable challenge for endophyte-related investigations. Morphologically, many culturable endophytic fungi fail to sporulate and are generally referred to as mycelia sterilia (Cui et al., 2021). Phylogenetically, during studies of endophytic fungi as sources of bioactive metabolites, molecular identification is typically performed using the ITS sequences or GenBank BLAST search (Raja et al., 2017; Zhang X. G. et al., 2020). However, the two approaches have distinct limitations, because they may not be able to distinguish some members with close phylogenetic relationships of certain genera (Oloo et al., 2019), and more than 1/4 GenBank fungal ITS sequences were submitted without sufficient taxonomic confirmation (Raja et al., 2017). Therefore, in this study, molecular identification was carried out using protein-coding and ribosomal genes, mainly including LSU, ITS, and TUB. Nevertheless, molecular phylogeny combined with morphology for species identification is still necessary, such as *Diaporthe* sp. 1 HGUP191027, *Coniella* sp. 1 HGUP190059, and *Thelonectria* sp. HGUP190169, possibly representing new taxa.

Sordariomycetes is one of the main dominant classes of fungal endophyte communities (Wang et al., 2019; Ettinger and Eisen, 2020). In this study, 242 isolates of endophytic Sordariomycetes were collected and identified from *R. roxburghii* in total, including 33 genera. The dominant genera were *Diaporthe* (31.4%), *Fusarium* (14.4%), *Chaetomium* (7.9%), *Dactylonectria* (7.0%), *Graphium* (4.5%), *Colletotrichum* (4.1%), and *Clonostachys* (4.1%). Similar results were observed by Fang et al. (2019) in a study of the diversity of endophytic fungi from *Ageratina adenophora*, where the dominant genera of cultivable endophytic fungi, belonging to the class Sordariomycetes, were *Diaporthe*, *Fusarium*, and *Colletotrichum*. These fungal genera are common endophytic fungi from different regions and plants around the world (Paul et al., 2014; Zhu H. et al., 2019; Du et al., 2020).

Alpha diversity applied to analyze species diversity was described by the Shannon-Wiener, Simpson, Margalef, and Pielou evenness index in this work. The results revealed that the diversity of endophytic Sordariomycetes from different tissues decreased in the order of root, fruit, stem, flower, leaf, and seed. Among the endophytic fungi of *Panax ginseng* Meyer, the diversity index and richness of the root were higher than stem and leaf tissues (Park et al., 2017). Nevertheless, the highest diversity indexes and species richness were obtained in the stem of *Zanthoxylum bungeanum* (Li et al., 2016). Moreover, diversity and species richness was analyzed statistically and found to be higher in leaf than in stem segments of three medicinal plants *Terminalia pallida*, *Rhynchosia beddomei*, and *Pterocarpus santalinus* (Venkateswarulu et al., 2018). According to these reports, the differences of endophytic communities in plant tissues are shaped by the factors such as plant species, soil type, geographic, and environmental conditions (Xie et al., 2020). In this study, endophytic fungi, *Clonostachys* and *Dactylonectria*, also exhibited obvious tissue specificity, which may be caused by differences in the plant tissue microenvironment or the different physiology and chemistry of the colonized tissues (Li et al., 2016). Additionally, functional annotation of fungi at the genus level indicated that fungi of the same genus have multitrophic lifestyles combining endophytic, pathogenic, and saprophytic behavior. Presumably, this is because many fungi may adapt their lifestyles to various changes in host and environmental conditions by evolving into endophyte-pathogen-parasites (Zhou et al., 2018).

Antimicrobial activity, one of the important biological activities of endophytic fungi, could largely be seen as the application potential in the field of plant disease control and pharmaceutical development (dos Santos et al., 2015; Li et al., 2016). Screening for antimicrobial activity is necessary because their natural compounds possess excellent activity against plant, animal, and human pathogens (dos Santos et al., 2015; Deshmukh et al., 2018). In the present work, 242 isolates of endophytic Sordariomycetes were subjected to primary screening for antimicrobial activity. Then, 40 strains with antimicrobial potential were further examined, and intracellular and extracellular metabolites were prepared, respectively. In re-screening, the results suggested that extracellular metabolites may be more potential than intracellular metabolites in the development of antimicrobial agents, which is probably since most of the intracellular metabolites are not adequately secreted and released (Zhu J. et al., 2019). The results of antimicrobial assays demonstrated that there were 11 isolates with good activities, for example, *Clonostachys rhizophaga* HGUP190070, *Coniella* sp. 2 HGUP196011, *Chaetomium globosum* HGUP190087, *Chloridium aseptatum* HGUP190073, *Diaporthe chimonanthi* HGUP191001, *D. eres* HGUP192111, *D. caryae* HGUP191078, *Fusarium sp.* HGUP190163, *Hypomontagnella monticulosa* HGUP194009, *Pseudothielavia arxii* HGUP190099, and *Nigrospora sphaerica* HGUP191020. Among these, *Hypomontagnella monticulosa* with antibacterial activity has been described by Lutfia et al. (2021). *Clonostachys rhizophaga* has been reported to possess antifungal activity (da Silva et al., 2021). *Chaetomium globosum* (Kaur and Arora, 2020), *Nigrospora sphaerica* (Wu et al., 2018), and some *Fusarium* spp. (Liang et al., 2016) have previously been reported in several studies to have antimicrobial activity *in vitro*. To date, however, there are only a few reports on the antimicrobial activities of the other six species. From the perspective of the broad spectrum and strength, *H. monticulosa* HGUP194009 and *N. sphaerica* HGUP191020 were considered as isolates with great antimicrobial potential. Thus, the present results were of great promise for the development of antimicrobial agents.

Finally, we also noted that *N. sphaerica* was a fungus that caused disease on numerous plants, e.g., blueberry, tea, and palm (Wright et al., 2008; Abass et al., 2013; Dutta et al., 2015). However, in the present study and some previous studies (Wu et al., 2018), *N. sphaerica* exhibited strong antimicrobial activities. Furthermore, *N. sphaerica* also exhibited anti-cancer, anti-inflammatory, and α-glucosidase inhibitory activities (Ukwatta et al., 2019). Therefore, some fungi that can cause disease may have beneficial biological activities as endophytic fungi.

## Conclusion

In this work, with six different media, 242 strains of endophytic Sordariomycetes were isolated from *R. roxburghii* and identified by multigene phylogenetic analysis. *Colletotrichum*, *Clonostachys*, *Chaetomium*, *Diaporthe*, *Dactylonectria*, *Fusarium*, and *Graphium* were found to be the dominant genera. The highest diversity indexes and species richness were obtained in the root tissues. Functional annotation of fungi at the genus level indicated that the same genus has multitrophic lifestyles, ranging from endophytic, saprotrophic to pathogenic. *In vitro* antimicrobial experiments showed that the extracellular metabolites of 11 isolates have good antimicrobial activities, especially *H. monticulosa* HGUP194009 and *N. sphaerica* HGUP191020. All of our results suggested that endophytic Sordariomycetes from *R. roxburghii* are potential sources for exploring antimicrobial agents.

## Data Availability Statement

The data presented in this study are deposited in the GenBank repository, accession numbers ITS: MZ724686–MZ724926; LSU: MZ724927–MZ725010; and TUB: MZ723979–MZ724131.

## Author Contributions

All authors listed have made a substantial, direct, and intellectual contribution to the work, and approved it for publication.

## Conflict of Interest

G-CT was employed by Food Safety and Nutrition (Guizhou) Information Technology Co., Ltd. The remaining authors declare that the research was conducted in the absence of any commercial or financial relationships that could be construed as a potential conflict of interest.

## Publisher’s Note

All claims expressed in this article are solely those of the authors and do not necessarily represent those of their affiliated organizations, or those of the publisher, the editors and the reviewers. Any product that may be evaluated in this article, or claim that may be made by its manufacturer, is not guaranteed or endorsed by the publisher.

## Footnotes

1
http://funguild.org
